# Health Tract, No. 122

**Published:** 1865

**Authors:** 


### HEALTH TRACT, No. 122.

### HOUSEWIFERY.

The earlier the breakfast, the more work will be got through with during the day, and the better health will the whole household have, because food or warm drink in the stomach antagonize the disease engendering damps, fogs, and miasms, which impregnate the air about sunrise, in all countries, especially in warm weather.

Quinces baked in sugar and water, or syrup, or simply baked and eaten with powdered sugar, make a good substitute for baked apples.

Potatoes may be kept a very long time from rotting, in a cellar protected against frost, by dusting the floor or bin with lime; then put down a layer of potatoes six inches thick; then dust with lime, another layer of potatoes, etc. One bushel or more of lime to forty of potatoes; they sprout least in darkness.

Woolen Flannel is the best protection against taking cold, in all seasons, if kept pliable by washing it in strong, hot soap-suds, without wringing, merely squeeze, then rinse in clear, warm water, and hang on a line to drip dry.

Silks are best, next the skin, for some persons. Wash them by spreading on a board smoothly; rub on white soap; brush with a hard brush, then brush off with cold water, applied to both sides. A little alum in the last water prevents colors from "running." Grease-stains are removed from silks by using equal parts of alcohol and camphene; never wring silk after washing, because the creases thus made will always remain. While "burning-fluid," which is a mixture of alcohol and turpentine, removes grease and other stains from light-colored silks and gloves, sour milk is good for bleaching linen; but grease is best removed from carpets with strong, cold soap-suds, thus avoiding the danger of camphene. Life has been lost by keeping oxalic acid in the house, to remove ink and iron stains; but as it is only suitable for white fabrics, (it should be plainly labeled and marked "Poison," in large letters, if kept about the house,) it is better to use the juice of lemons or of sorrel leaves, especially as the oxalic acid eats the fabric, unless immediately and thoroughly washed off.

Persons have been suffocated by inhaling the fumes of burning sulphur, when used to bleach out colors and stains of fruits and vegetables particularly, hence the fumes should be conveyed to the stained spot by means of a funnel-shaped paper roll; but it is safer to dip stained fabrics in sour milk, then dry in the sun, repeating the operation until the bleaching is perfected,

Flannel Shirts, or other woolens, should have grease spots removed without fulling them up, thus: Put one ox-gall in three gallons of cold water, in which immerse the garment, and squeeze or pound (not wring) it, until the spots are removed; then thoroughly wash in cold water, else the odor of the gall becomes very disagreeable.

If burning-fluid or benzole are used to remove grease or other stains, let it be at least two yards from any blaze of candles, gas, lamp, or fire. Valuable lives are lost every year by neglecting this precaution.

Eggs are good which are diaphanous, or show a faint reddish color, when held in a dark place, toward a candle or other light, when held in the circle made by the thumb and forefinger; they are bad in proportion as they seem black. This is an infallible test.

Milk is kept good longer, if it is boiled, evaporated, condensed, or kept still at a temperature of about forty degrees. If heated three days in succession in summer, and two in winter, (as per Guy Lussac's experiments,) up to the boiling-point, it will keep two months without souring.

The best way of keeping milk in summer, is to have a spring-house well shaded, and on the north side of a hill, the pans sitting in a stream of running water, protected against currents of air. The country people deliver milk at the railroad for two cents a quart, one cent freight to the city, where it is delivered at sunrise to our citizens for seven cents a quart, or six cents at 146 East Tenth street.

Peaches are peeled without waste, when fully ripe, by pouring boiling water on them, and let them remain a minute, to cook only skin-deep, as, in tomatoes.

Clinkers are removed from stove-grates and range-backs, thus: When the coal is all aglow, throw in half a dozen broken oyster-shells, cover these over with fresh coal, and when all are red-hot, the clinkers are doughy and are easily removed.

Carpet-Sweeping.—Draw the broom to you with short, quick strokes, taking up the dirt every half-yard, in a dust-pan, or at each stair, and thus avoid working the dirt into the cleaner parts. Never use tea-leaves, paper, or damp grass, to collect the dust, let the dust-pan do that.

Weather-wise.—Allow the sugar to dissolve in your coffee or tea without stirring; if froth remains in the center, durable fine weather is indicated; but rainy if it settles around the sides; variable if it remains between the two, so says M. Sauvageon.

